# Evidence for the expression of TRPM6 and TRPM7 in cardiomyocytes from all four chamber walls of the human heart

**DOI:** 10.1038/s41598-021-94856-4

**Published:** 2021-07-29

**Authors:** Inga Andriulė, Dalia Pangonytė, Mantė Almanaitytė, Vaiva Patamsytė, Milda Kuprytė, Dainius Karčiauskas, Kanigula Mubagwa, Regina Mačianskienė

**Affiliations:** 1 Institute of Cardiology, Lithuanian University of Health Sciences, Kaunas, Lithuania; 2 Department of Cardiac, Thoracic and Vascular Surgery, Hospital of Lithuanian University of Health Sciences Kauno Klinikos, Lithuanian University of Health Sciences, Kaunas, Lithuania; 3 Department of Cardiovascular Sciences, Faculty of Medicine, KU Leuven, Leuven, Belgium; 4 Department of Basic Sciences, Faculty of Medicine, Université Catholique de Bukavu, Bukavu, DR Congo

**Keywords:** Cell biology, Molecular biology

## Abstract

The expression of the channels-enzymes TRPM6 and TRPM7 in the human heart remains poorly defined, and TRPM6 is generally considered not to be expressed in cardiomyocytes. We examined their expression at protein and mRNA levels using right atrial samples resected from patients (n = 72) with or without ischemic heart disease (IHD) and samples from all chamber walls of explanted human hearts (n = 9). TRPM6 and TRPM7 proteins were detected using immunofluorescence on isolated cardiomyocytes, ELISA on tissue homogenates, and immunostaining of cardiac tissue, whereas their mRNAs were detected by RT-qPCR. Both TRPM6 and TRPM7 were present in all chamber walls, with TRPM7 being more abundant. TRPM6 was co-expressed with TRPM7. The expression levels were dependent on cell incubation conditions (presence or absence of divalent cations, pH of the extracellular milieu, presence of TRP channel inhibitors 2-aminoethoxydiphenyl-borate and carvacrol). These drugs reduced TRPM7 immunofluorescence but increased that of TRPM6. TRPM6 and TRPM7 expression was increased in tissues from IHD patients. This is the first demonstration of the presence and co-expression of TRPM6 and TRPM7 in cardiomyocytes from all chamber walls of the human heart. The increased TRPM6 and TRPM7 expression in IHD suggests that the chanzymes are involved in the pathophysiology of the disease.

## Introduction

TRPM7 and its homologue TRPM6 belong to the TRP channel melastatin subfamily, are regulated by intracellular magnesium and are distinguished from other ion channels by unusual bifunctional activities, ion channel and protein kinase^1–3^. Both of them are permeable to Ca^2+^ and Mg^2+^, and are key regulators of Mg^2+^ homeostasis, but their tissue expression levels appear to be different. Current evidence shows that TRPM7 is ubiquitously expressed in all mammalian cells, with the highest expression in the heart and kidney^3,4^, whereas TRPM6 expression is restricted to epithelial cells of the kidney, placenta and intestine^5,6^. They are reported to play a role in various physiological or pathological processes, including intracellular Ca^2+^ and Mg^2+^ homeostasis^7^, pace-making^8^, fibrosis and inflammation^9^, etc. The functional characterization of these channels has relied mainly on electrophysiological methods^10^, including in cardiac myocytes^11–14^. Except for a differential pharmacological sensitivity to some substances (e.g., 2-aminoethoxydiphenyl-borate (2-APB)), the biophysical properties of murine or human TRPM6 and TRPM7 constructs expressed in various cell lines are almost indistinguishable^10^. However, the electrophysiological characterization has been limited by the lack of specific blockers. In addition, in native cardiomyocytes the majority of TRPM6 and TRPM7 proteins appear to reside in intracellular vesicles^15,16^, and are therefore inaccessible to patch clamp recordings.

To date, studies of TRPM6 and TRPM7 channel expression either in un-diseased^17^ or diseased^18^ human heart has relied mainly on the genomic approach. However, data on *Trpm6* or *Trpm7* mRNA and protein expression in different parts of the heart has remained scant, and conflicting results have been reported, especially concerning TRPM6. In both animals and humans, whereas some studies found that *Trpm6* mRNA was undetectable in the heart^19–21^, one study identified *Trpm6* mRNA and protein in the human heart and showed their increase in right atrial cardiomyocytes from patients with atrial fibrillation (AF)^22^. In most others studies information of TRPM6 was not included^18,23^. Thus, the presence of TRPM6 in the heart remains controversial and, more specifically, whether it and TRPM7 are expressed in all chamber walls of the heart is an open question.

The present study used different approaches, including protein detection by immunostaining of isolated cardiomyocytes or of cardiac tissue, protein measurements by ELISA in cardiac homogenates and mRNA detection by real-time quantitative polymerase chain reaction (RT-qPCR), to examine TRPM6 and TRPM7 expression. The data confirm the presence of TRPM6 protein and gene alongside TRPM7 in the walls of all four chambers of the adult human heart. Moreover, we report on the modulation of the measured TRPM6 and TRPM7 fluorescence by the ionic composition of the cell incubation medium, by pharmacological drugs and by the pathological condition of ischemic heart disease.

## Results

### Immunofluorescence detection and co-expression of TRPM6 and TRPM7 in cardiomyocytes from the walls of all four chambers of the human heart

**Figure 1:**
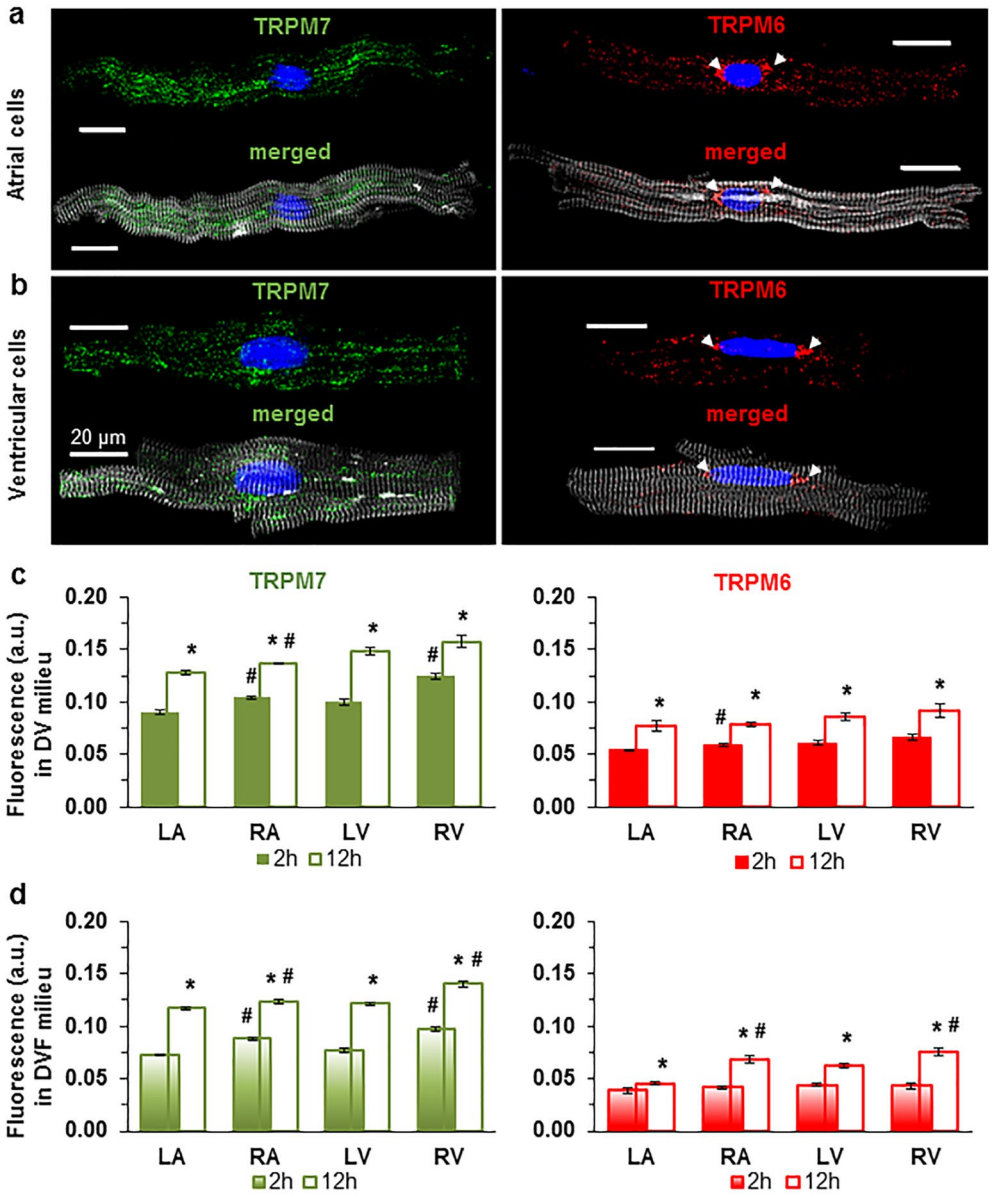
Immunofluorescence of TRPM7 and TRPM6 proteins in all cells used. Image acquisition performed using confocal laser scanning microscope (**a**, atria; **b**, ventricle). Immunofluorescence of confocal z-stack of cardiomyocytes with immunodetected TRPM7 and TRPM6 proteins, respectively. Alexa Fluor 488 and Alexa Fluor 546 for the TRPM7 and TRPM6 protein appear in green and red, respectively. Alexa Fluor 405 for F-actin cytoskeleton appears in surrogate grey. Hoechst 33342 for nuclei appears in blue (the arrowheads indicate the localization of TRPM6 protein in the perinuclear area). (**c**, **d**) Quantification of immunofluorescence levels of the TRPM7 (*green*) and TRPM6 (*red*) proteins in cardiomyocytes from four chambers of the heart (left atrium, LA; right atrium, RA; left ventricle, LV; and right ventricle, RV), under experimental conditions with (**c**) and without (**d**) divalent cations in the extracellular milieu, respectively. Cardiomyocytes were fixed following 2 h (*filled columns*) or 12 h (*unfilled columns*) after cell isolation. Mean data provided in arbitrary units (a.u.) (Supplementary Table 1 online). A blinded study-design (with the investigator reading the fluorescence not knowing the cell incubation conditions) was used for the detection of protein concentration during various experimental conditions. *P < 0.05 2 h vs. 12 h and #*P* < 0.05 right-sided vs. left-sided heart chambers. Scale bars indicate 20 µm.

We undertook to carry out an in-depth comparison of both TRPM7 vs. TRPM6 protein expression in the four cardiac chambers using several molecular approaches. First, we used the immunostaining of TRPM7 and TRPM6 proteins of atrial and ventricular cardiomyocytes, performed 2 h or 12 h after cell isolation. Figure 1 illustrates that all cardiomyocytes expressed both channels, with a lower expression level for TRPM6 (Fig. 1a,b). Noticeably, the immunofluorescence of TRPM6 protein, in contrast to TRPM7, appeared to be highest in the perinuclear area in about half cases (44.7% from ~ 400 cells; see Fig. 1a,b, arrows). This indicates that part of detected fluorescence is from the intracellular membranes, especially given the permeabilization with Triton-X. TRPM7 and TRPM6 protein expression was slightly but significantly higher for the right-sided vs. left-sided chambers (Fig. 1c,d; see also Supplementary Table S1 online). The expression level for either TRPM6 or TRPM7 was higher when cells were fixed after 12 h of cardiomyocyte conservation (Fig. 1c,d; compare filled and unfilled columns, respectively). In general, the immunofluorescence level of both channel proteins in cardiomyocytes from all chamber walls was significantly higher following cell incubation in divalent cation-containing (DV) extracellular conditions vs. following incubation in divalent cation-free (DVF) conditions (Fig. 1c vs. d).

**Figure 2:**
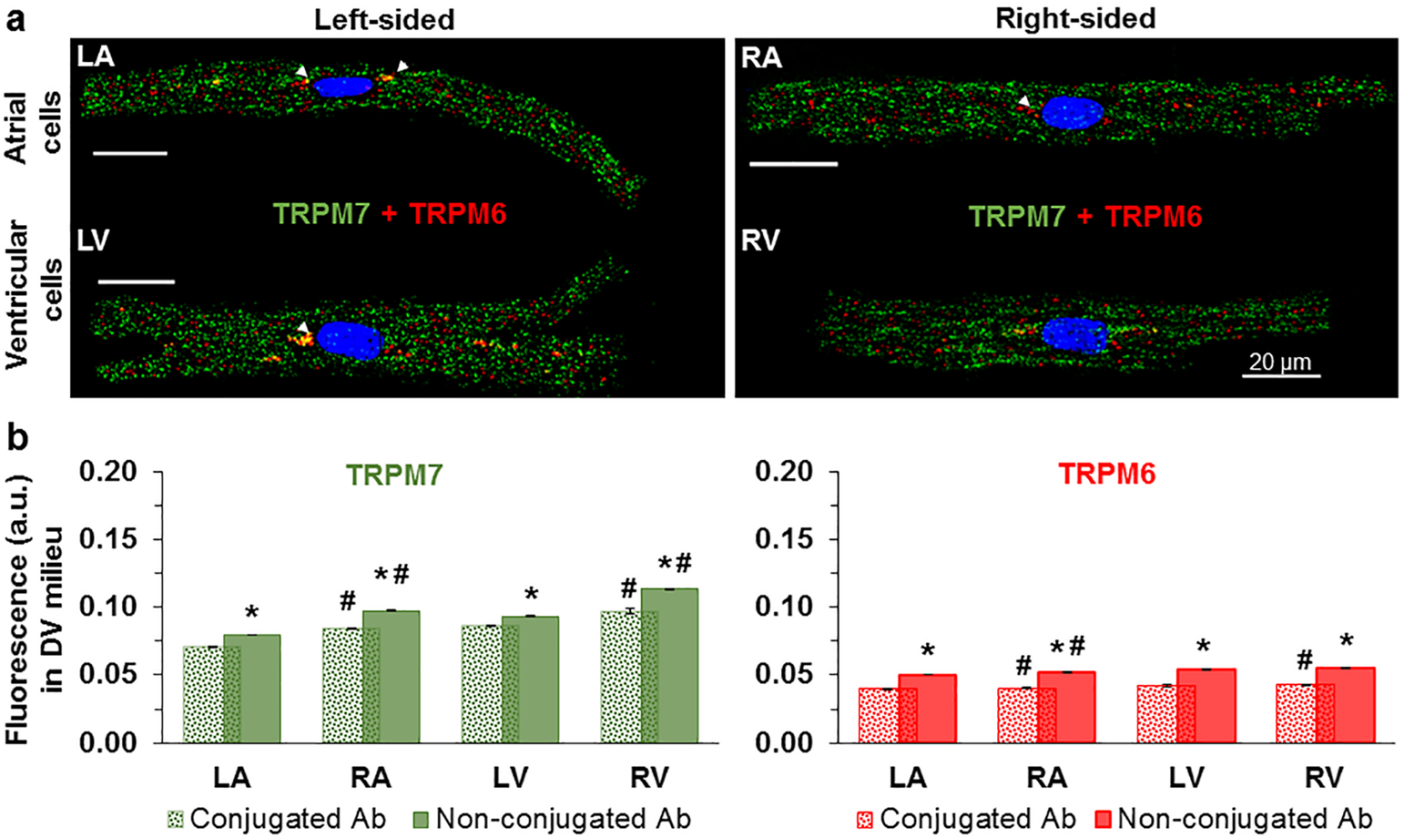
Immunofluorescence images depicting co-expression of TRPM6 and TRPM7 proteins in human cardiomyocytes. (**a**) The immunofluorescence of TRPM7 (*green*) and TRPM6 (*red*) in the same LA, RA, LV, and RV cardiomyocyte when using conjugated antibodies (the arrowheads indicate the localization of TRPM6 protein in perinuclear area). (**b**) Quantification of the staining intensity of the immunodetected conjugated antibodies (*spotty*) and non-conjugated antibodies (*smooth*) for both proteins in cardiomyocytes from the four chambers of the heart as indicated. *P < 0.001 2 h vs. 12 h, #*P* < 0.001 right-sided vs. left-sided heart chambers. Other notations are the same as in Fig. 1.

Figure 2 illustrates immunofluorescence images obtained using conjugated primary antibodies to simultaneously detect both TRPM6 and TRPM7 proteins expression in the same cell. The data indicate a co-expression of TRPM6 and TRPM7 proteins in cardiomyocytes from the four heart chambers (Fig. 2a). Like with non-conjugated antibodies, levels of TRPM7 were higher vs. those of TRPM6 in all chamber walls. The fluorescence level with conjugated antibodies for both channels was lower compared with that of the non-conjugated primary antibodies (compare Fig. 2b with Fig. 1b, see also Supplementary Tables S1, S2 online). Altogether, the above results provide a consistent evidence for a co-expression of both TRPM6 and TRPM7 in cardiomyocytes from the four chamber walls of the human heart.

### Opposite effects of 2-APB and CAR on immunofluorescence of TRPM6 and TRPM7 in the human heart

**Figure 3:**
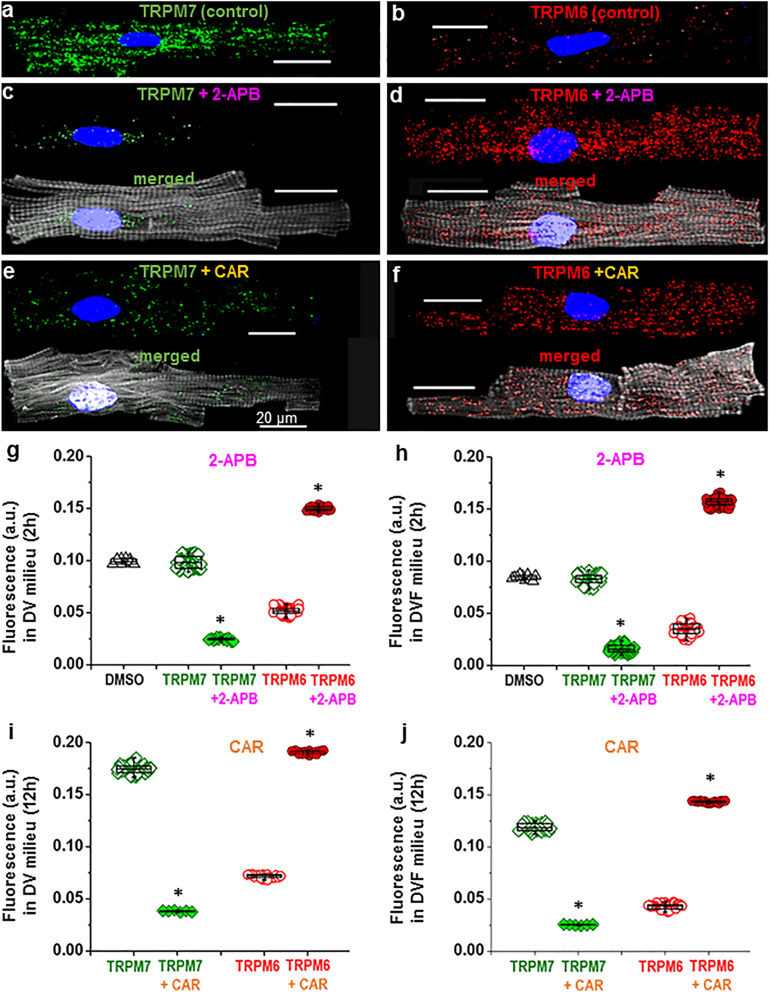
Effect of 2-APB and CAR on the immunofluorescence of TRPM6 and TPRM7 in human cardiomyocytes. (**a**–**f**): Cardiomyocyte staining with anti-TRPM7 (**a**, **c**, **e**) or anti-TRPM6 (**b**, **d**, **f**) in the absence (**a**, **b**) of drugs and in the presence of either 2-APB (**c**, **d**) or CAR (**e**, **f**). (**g**–**j**): Quantification of the intensity of fluorescence without drugs (*open*) and with the drugs (*filled*) expressed in arbitrary units (a.u.). Note lack of influence of the solvent, DMSO, at 500 μmol/L (triangles) but opposite change with 2-APB and CAR on TRPM7 vs. TRPM6, i.e. decrease of the immunofluorescence level of TRPM7 but increase of the TRPM6 fluorescence level. *P < 0.001 drug vs. no drug. Other notations are the same as in Fig. 1.

In addition to the presence/absence of extracellular divalent cations, the presence of pharmacological drugs known to inhibit TRPM6 and TRPM7 channel function also had an effect on the immunofluorescence levels of TRPM6 and TRPM7. In our study the cardiomyocytes were exposed to 2-APB (500 μmol/L) or CAR (100 μmol/L), added for 15 min after keeping the cells for 2 h or 12 h, but before cell fixation and subsequent incubation with primary antibodies. Figure 3a,b illustrates immunostaining images of TRPM7 (*green*) and TRPM6 (*red*) proteins under control conditions (i.e., without the drugs) as well as after pre-treatments with 2-APB (Fig. 3c,d) or CAR (Fig. 3e,f). Cells treated with these drugs displayed weaker TRPM7 fluorescence intensity but stronger TRPM6 intensity. Mean fluorescence levels are presented in Fig. 3g,h, respectively. Whereas the vehicle, DMSO, did not change fluorescence intensity (0.0993 ± 0.0008 a.u. and 0.0847 ± 0.0007 a.u. in solutions with and without divalent cations, respectively; n = 10–11), with 2-APB (500 µmol/L) immunodetected TRPM7 was decreased (from 0.0986 ± 0.0006 a.u. to 0.0246 ± 0.0003 a.u., n = 16–88, *P* < 0.001, in solutions with divalent cations, and from 0.0827 ± 0.0005 a.u. to 0.0162 ± 0.0004 a.u., n = 70–76, *P* < 0.001, in solutions without divalent cations). Under the same experimental conditions 2-APB (500 μmol/L) caused an increase in TRPM6 fluorescence (from 0.0518 ± 0.0003 a.u. to 0.1500 ± 0.0004 a.u., n = 20–99, *P* < 0.001, and from 0.0354 ± 0.0006 to 0.1569 ± 0.0005 a.u., n = 70–87, *P* < 0.001, in solutions with and without divalent cations, respectively). Qualitatively similar changes were caused by CAR in cardiomyocytes incubated for 2 h (not illustrated) or for 12 h before addition of CAR (Fig. 3i,j): TRPM7 fluorescence decreased from 0.1748 ± 0.0006 a.u. to 0.0383 ± 0.0003 a.u., n = 6–64, *P* < 0.001; and from 0.1188 ± 0.0007 a.u. to 0.0251 ± 0.0002 a.u., n = 6–32, *P* < 0.001, respectively. In contrast, TRPM6 fluorescence increased from 0.0717 ± 0.0004 a.u. to 0.1907 ± 0.0004 a.u., n = 16–20, *P* < 0.001, and from 0.0428 ± 0.0004 a.u. to 0.1433 ± 0.0002 a.u., n = 17–37, *P* < 0.001, respectively.

### Extracellular proton concentration affects the immunofluorescence of TRPM6 and TRPM7 in human cardiomyocytes

**Figure 4:**
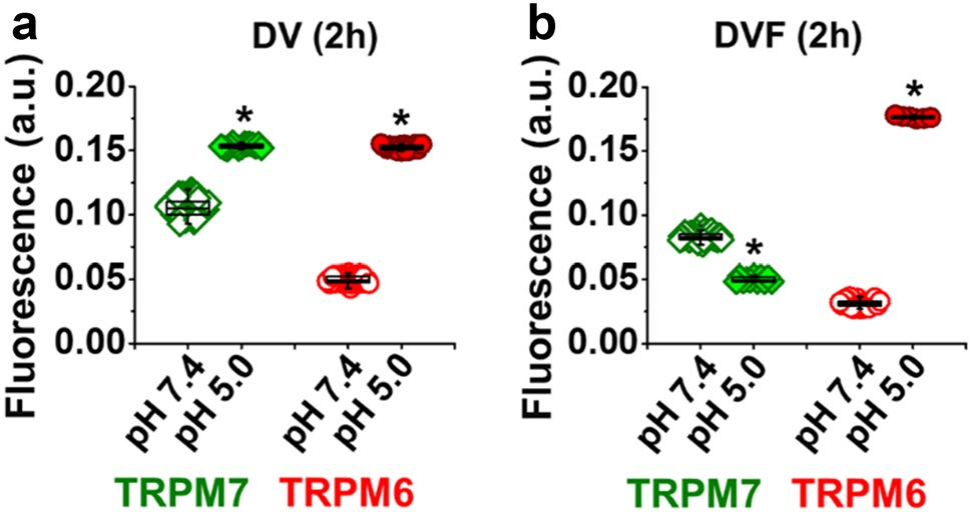
Effect on incubating human cardiomyocytes in acidic solution on the immunofluorescence of TRPM6 and TPRM7. (**a, b**) Quantification of the intensity of fluorescence at pH = 7.4 (*open symbols*) and at pH = 5.0 (*filled symbols*) expressed in arbitrary units (a.u.) with and without divalent cations, respectively. *P < 0.001 pH 5.0 vs. pH 7.4. Other notations are the same as in Fig. 1. Extracellular acidification differently changes the fluorescence level of the immunodetected TRPM7 (*filled diamonds*) and TRPM6 (*filled circles*) proteins in human cardiomyocytes depending on the presence of divalent cations in the milieu.

Like extracellular divalent cations and pharmacological agents, extracellular pH is known to modulate TRPM6 or TRPM7 channel function^11,12,14^. We also examined the possible impact of extracellular acidity on the immunofluorescence. Cardiomyocytes were pre-incubated for 2 h in an acidic extracellular milieu with and without divalent cations, just before fixation. As illustrated in Fig. 4a, extracellular acidification to pH 5.0 increased immunofluorescence level of TRPM7 and TRPM6 by 1.45 fold and 3.06 fold, respectively (to 0.1536 ± 0.0004 a.u. and to 0.1524 ± 0.0004 a.u., respectively; n = 15–25, *P* < 0.001), relative to the fluorescence detected in cells kept at pH 7.4 (0.1057 ± 0.0008 a.u. and 0.0498 ± 0.0003 a.u., respectively; n = 61–69, *P* < 0.001). In this case, an interaction was observed with extracellular divalent cations. Acidification to pH 5.0 during incubation without divalent cations changed the fluorescence level of immunodetected proteins differently, as presented in Fig. 4b: suppression of TRPM7 (from 0.0830 ± 0.0004 a.u. to 0.0500 ± 0.0006 a.u., n = 11–51, *P* < 0.001), but enhancement of TRPM6 by 5.62 fold (from 0.0314 ± 0.0005 a.u. to 0.1765 ± 0.0004 a.u., n = 6–25, *P* < 0.001). The data indicate that both immunodetected proteins are not coupled and show individual regulation under extracellular acidification of the milieu.

### Influence of underlying diseases on the immunodetected TRPM6 and TRPM7

**Figure 5:**
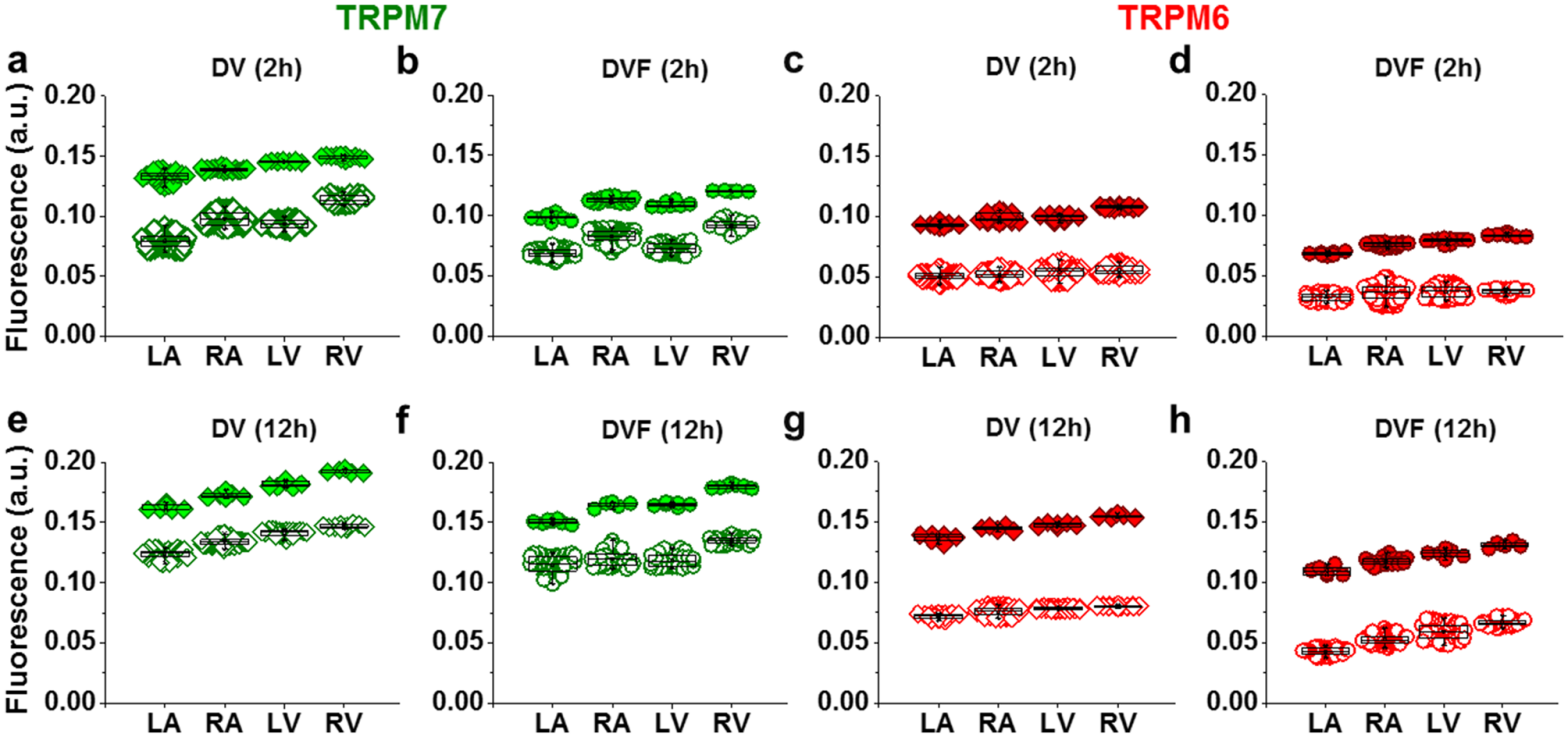
Comparison of the levels of TRPM6 and TRPM7 in IHD vs. non-IHD. (**a–d**, **e–h**) Quantification of the intensity of fluorescence in cardiomyocytes obtained from patients with IHD (*filled symbols*) and without such diagnosis (*unfilled symbols*) expressed in arbitrary units (a.u.) in presence/absence of divalent cations, 2 h and 12 h, respectively. In all cells used *P* < 0.001 IHD vs. non-IHD. Other notations are the same as in Fig. 1. IHD increases the fluorescence level of the immunodetected TRPM7 (*green*) and TRPM6 (*red*) proteins expression.

Since our previous electrophysiological studies indicated an influence of IHD on the TRPM7 current density^12^, we also sought to determine whether such a pathological condition also influences the levels of immunodetected TRPM6 and TRPM7 proteins. Figure 5 compares the mean levels of TRPM6 and TRPM7 protein immunostaining in cardiomyocytes from patients with or without a clinical history of IHD. In general, independently of the experimental conditions used to incubate cells (i.e. presence/absence of extracellular divalent cations, 2 h or 12 h incubation), the immunofluorescence levels of both channel proteins were much higher in cardiomyocytes of the IHD patient group compared to those from non-IHD patients (Fig. 5, upper values of each panel; see also Supplementary Tables S3, S4 online).

### Validations of TRPM6 and TRPM7 proteins expression by ELISA and of their mRNA expression by RT-qPCR in the four chambers of the human heart

**Figure 6:**
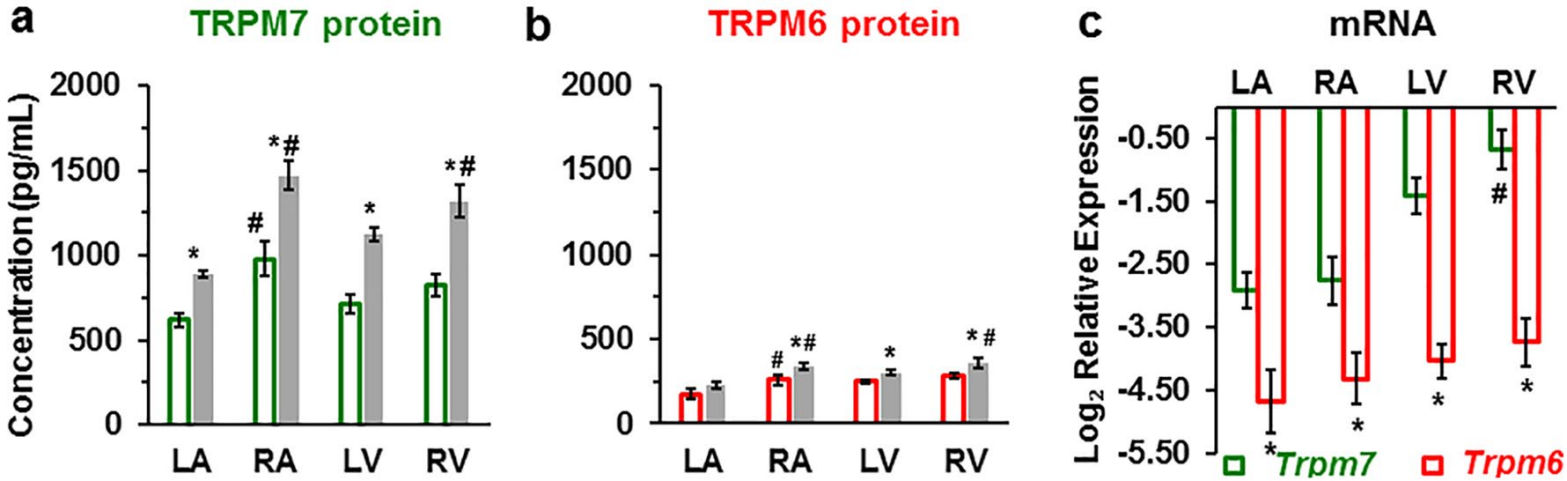
TRPM7 and TRPM6 protein levels and RT-qPCR mRNA relative expression levels in human heart tissue homogenates. (**a, b**) TRPM7 and TRPM6 proteins are increased in the walls of all heart chambers with IHD (*filled columns*) vs. non-IHD (*unfilled columns*). A blinded study-design (with the diagnosis unknown to the investigator) was used for the detection of protein concentration in the various samples. Values (mean ± SEM) are in pg/mL and from 3–33 heart tissue homogenates. *P < 0.05 IHD vs. non-IHD, #*P* < 0.05 right-sided vs. left-sided using ANOVA with LSD test (Least Significant Difference). (**c**) mRNA relative expression levels of human *Trpm6* and *Trpm7* genes detected in the four cardiac chambers from explanted human hearts (n = 3, for each). *P < 0.05 *Trpm7* vs. *Trpm6*, #*P* < 0.05 right-sided vs. left-sided.

Having characterized TRPM6 and TRPM7 expression using immunofluorescence, we wanted to examine whether the results can be validated using a different technique, ELISA carried on tissue homogenates. Figure 6a,b illustrates that ELISA also detected the presence of TRPM7 and TRPM6 proteins in all cardiac chamber walls. The results are qualitatively similar to those obtained using immunofluorescence: higher expression of TRPM7 compared to TRPM6 (Supplementary Table S5 online), higher expression in right chambers compared to left chambers, increased expression in IHD. We also found that in the LV the expression of the TRPM7 and TRPM6 proteins was significantly (*P* < 0.05) higher in subepicardial homogenates (1313.75 ± 15.81 pg/mL and 324.33 ± 22.22 pg/mL, respectively; n = 3) compared with subendocardial homogenates (1114.80 ± 23.96 pg/mL and 256.76 ± 19.24 pg/mL, respectively; n = 3).

Figure 6c shows the results of RT-qPCR to assess TRPM6 and TRPM7 expression at the mRNA level using primers designed for the human genes (*Trpm6* and *Trpm7*). *β-actin* (ACTB) was used as internal control. Both *Trpm6* and *Trpm7* mRNAs were detected in cardiac tissues from explanted human hearts. Both had 100% qPCR efficiency for all four cardiac chamber walls (Fig. 6c). Here also RT-qPCR showed that the *Trpm6* gene relative expression was much lower compared to the *Trmp7* gene expression (Fig. 6c, Supplementary Figure S1, and Supplementary Table S6 online). *Trpm7* mRNA expression was more than two- to fourfold higher in ventricular tissue compared to atria (Fig. 6c, *green*). In general, RT-qPCR results are in agreement with the expression of the proteins. However, in contrast to results with immunofluorescence, there was no significant difference of the *Trpm6* mRNA expression level in right vs. left sides.

### Demonstration of TRPM6 and TRPM7 expression by immunohistochemistry

**Figure 7:**
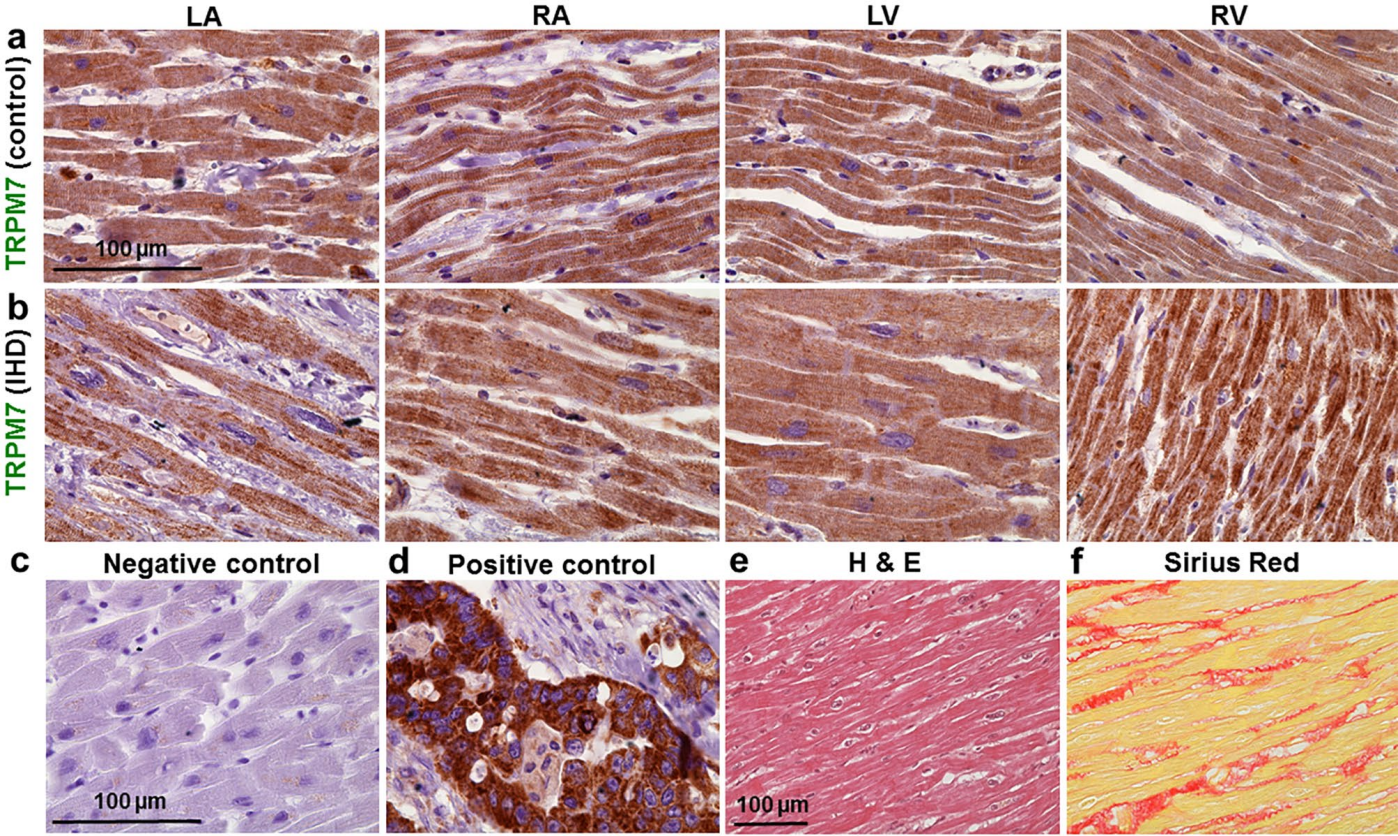
Representative histotopograms of TRPM7 images in the four chamber walls of the human heart. (**a**) Control subject after traffic accident. (**b**) IHD patient. The intensity of the brownish pigments shows areas with expression of TRPM7 in the heart (× 40 magnification). (**c**) Negative control in the LV. (**d**) Positive control of TRPM7 in tumour of the colon (**a–d**, × 40 magnification). Note: for negative control an irrelevant IgG of the same isotope as the primary antibody was used. (**e**,**f**) Representative images of the regional assessment (from the LV midmyocardial tissue sections) of myocardial hypertrophy/degeneration and fibrosis using hematoxylin and eosin (H & E) staining and picrosirius red staining, respectively (× 20 magnification). Scale bars indicate 100 µm.

**Figure 8:**
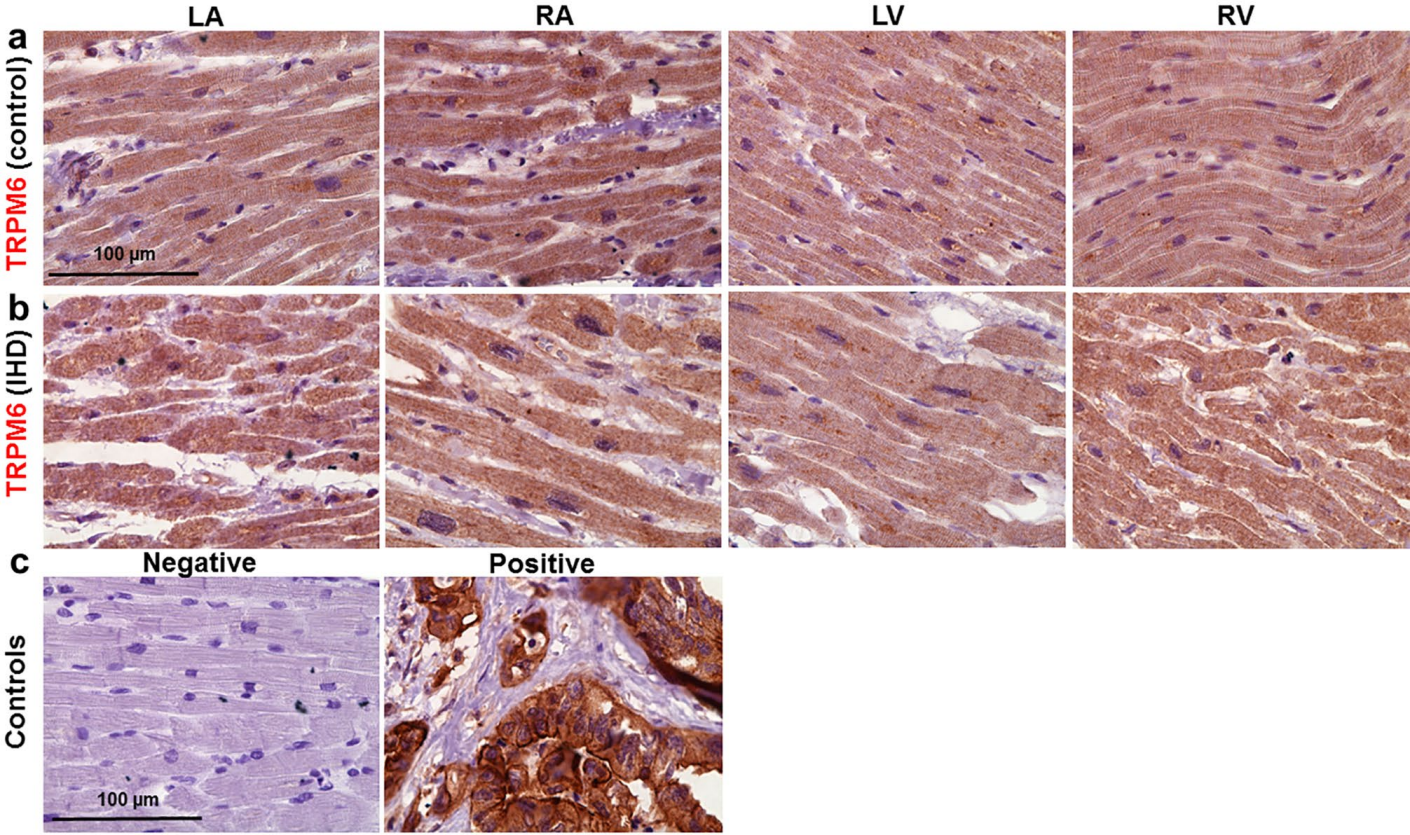
Representative histotopograms of TRPM6 images in the four chamber walls of the human heart. (**a**) Control subject after traffic accident. (**b**) IHD patient. The intensity of the brownish pigments shows areas with expression of TRPM6 in the heart (× 40 magnification). (**c**) Negative control (*left*) in the LV, and positive control (*right*) of TRPM6 in tumour of the colon (× 40 magnification). Scale bars indicate 100 µm. Other notations are the same as in Fig. 7.

Finally, to further demonstrate TRPM6 and TRPM7 expression in the human heart, histologic preparations of ventricular and atrial tissues were exposed to primary antibodies and stained with Mayer’s hematoxylin. Color intensity analysis of TRPM7 (Fig. 7) as well as of TRPM6 (Fig. 8) in tissues showed noticeable brownish pigments in the intracellular space in tissues from the four chamber walls of non-ischemic heart (obtained from a victim of traffic accident and serving as control; Figs. 7a,8a) and of an IHD heart (Figs. 7b,8b). Morphological changes of myocardial tissues from the IHD patients included cardiomyocyte hypertrophy and nuclear enlargement (see also Fig. 7e) as well as interstitial fibrosis (Fig. 7f). Qualitatively similar data on TRPM6 and TRPM7 staining were obtained in heart tissue sections from all subjects of this study (Supplementary Table S7 online).

## Discussion

Our study provides evidence of cardiac co-expression of TRPM6 and TRPM7 in cardiomyocytes from all four chamber walls of the human heart. Till now there has been consistent evidence for the expression of TRPM7 in cardiac tissue, including in contractile (atrial and ventricular) cardiomyocytes and conduction cells as well as in fibroblasts (see^24^). TRPM7 is likely also expressed in coronary vascular endothelial and smooth muscle cells like in peripheral vasculature^25^. But controversy exists concerning the expression of TRPM6. In addition, investigation on TRPM6 and TRPM7 expression in all four chamber walls of the human heart was still lacking. Here, we employed different approaches to examine this expression. First, using immunofluorescence on cardiomyocytes isolated from any part of the human heart, we detected expression of TRPM6 alongside with TRPM7 expression. In support of these results, TRPM6 and TRPM7 proteins were also detected by immunohistochemistry, they were detected and quantified by ELISA and their corresponding mRNAs were identified by RT-qPCR. The TRPM7 expression level was consistently much higher than that of TRPM6. Thus, our data provide for the first time evidence of the expression of both channels in the walls of all four human heart chambers.

Information regarding the TRPM6 channel protein level in the human heart has remained scant, and has concerned only RA tissues^22^. Information regarding the *Trpm6* mRNA level in human RA has also been controversial, since in two studies no mRNA expression of *Trpm6* was detected^14,20^, whereas in another study *Trpm6* mRNA was present and its expression found to be increased in RA cardiomyocytes from patients with AF^22^. There is no previous report showing the presence of TRPM6 in ventricular cells or tissues. In LV and RV tissues obtained from patients with advanced heart failure (HF)^26^ or various mouse strains *Trpm6* channel gene was not detectable^21^.

The present study also provides information of the relative expression of the channels when comparing atrial vs. ventricular cells, or left vs. right cavity walls. TRPM6 or TRPM7 protein expression was of the same order of magnitude in atrial and ventricular cells, but expression was higher on the right side than on the left side. In the study by Morine and colleagues (2016)^26^, while failing to detect *Trpm6* and *Trpm7* mRNA in LV or RV from human subjects with end-stage HF, they were however able to show a biventricular expression of both channel mRNA in a mouse model of thoracic aortic constriction. A very recent study showed various gene expressions in the four chambers of the mouse hearts^27^, and demonstrated that *Trpm4* and *Trpm7* are predominantly expressed at the atrial level. It should be noted that gene detection is likely to be less specific than the other methods (cell immunostaining and ELISA) for determining levels of expression in cardiomyocytes due to contamination by the numerically more abundant non-cardiomyocyte cells (endothelial and vascular smooth muscle cells as well as fibroblasts).

We have no explanation for the different levels in the various chamber walls. Because TRPM channels are stretch sensitive, one possibility could be that expression in the various chamber walls is related to stretch. Ventricular cells, which experience more stress are shown in the present study to have slightly higher TRPM7 and TRPM6 expression although, as stated above, the difference with levels in atrial cells did not reach statistical significance. However, right cardiac chamber walls where mechanical stress in expected to be lower than left chamber walls display higher TRPM7 and TRPM6 levels. If stretch was the main factor determining TRPM7 or TRPM6 expression, then the following order would be expected in the levels of expression: LV, RV, LA and RA.

TRPM6 and TRPM7 can form homomers but can also heteromerize^28–30^ to form TRPM6/TRPM7 complexes. There are differences in ion permeability, single channel conductance and sensitivity to modulators between the various homo- and heteromers^10,30,31^. The different levels of expression of the two proteins, and the fact that their expression could be regulated in opposite directions (decrease of one vs. increase of the other; see below) indicate that these channels are likely to form homomers in addition to forming heteromers in cardiac myocytes. The possibility of being regulated in opposite or same directions is also good evidence that the antibodies used, directed towards these two channels, did not display promiscuous sensitivity to the two proteins.

Beside providing evidence for the presence of both TRPM6 and TRPM7, our data also show that experimental conditions such as the presence/absence of extracellular divalent cations, the presence of TRPM6 and TRPM7 channel current inhibitors 2-APB and CAR, and acidification of the cell incubation medium all cause a change in immunodetected channel protein levels. All these factors have been shown in electrophysiological studies to alter TRPM6 and TRPM7 channel current^10,13,32,33^.

The effects of extracellular divalent cations in our study (decreased expression of TRPM6 and TRPM7 in cells incubated in the absence of extracellular divalent cation; see Figs. 1c,d and 3g–j) is opposite to the one observed in previous studies using other cell types. Incubating in media containing low concentrations of divalent cations led to an upregulation of TRPM7 gene expression in chondrocytes^34^. Similarly, incubation in low extracellular Ca^2+^ and Mg^2+^ led to an increased expression of TRPM7 but not of TRPM6 in osteoblast-like cells^35^, and incubation in low-Mg^2+^ media led to an upregulation of TRPM6 in mammary epithelial cells^36^. Given the role of TRPM6/7 as pathways for the entry of Ca^2+^ and Mg^2+^, these changes can be interpreted as adaptive processes, initiated in order to compensate for the absence or decreased availability of the permeant divalents. It remains unclear why in our study TRPM6 and TRPM7 were downregulated instead of being upregulated in cells incubated in the absence of divalent cations, but other changes induced by the absence of divalent cations, e.g. alterations of intracellular ions such as Na^+^ (which increases in the absence of extracellular divalents^37^) or of signaling pathways linked to the functioning of these channels could add to and distort the above-mentioned adaptive process.

Although the present study did not investigate the effect of incubating cardiomyocytes in low or high Mg^2+^, others have noted that low extracellular Mg^2+^ changes the levels of TRPM7 in vascular smooth muscle and endothelial cells. For example, TRPM7 (but not TRPM6) was shown to be upregulated in human umbilical vein endothelial cells (HUVEC)^38^. These changes were interpreted as being adaptive, as explained above. However, opposite results, i.e. downrelgulation of TRPM7 was obtained in human microvascular endothelial cells (HMVEC)^39^. At present, like for the opposite effects with extracellular Ca^2+^, there is no definitive explanation for these differences, except for different involvements of oxidative stress, which is increased by low Mg^2+^ in HUVEC but not in HMVEC^38^. As far as cardiomyocytes are concerned, it should be noted that they differ from endothelial cells in that they are not actively proliferating. Thus, changes of intracellular ion homeostasis may subserve different purposes compared to endothelial cells where cell proliferation is very intense.

In our study, 2-APB and CAR, two pharmacological agents known as modulators of TRP channel activity, caused opposite effects on the fluorescence intensity of immunodetected TRPM7 and TRPM6 proteins. We intentionally used 500 μmol/L 2-APB since such a concentration has been demonstrated to differently regulate TRPM6 and TRPM7 channels: significant increase of the TRPM6 current^10^ vs. dramatic reduction of the TRPM7 current amplitude^40^. Our data of the influence of 2-APB on the fluorescence of immunodetected TRPM6 and TRPM7 proteins are in concordance with these electrophysiologic reports of opposite effects on the channels. Interestingly, CAR also caused quantitatively similar reduction of the fluorescence of the immunodetected TRPM7 but marked enhancement of the TRPM6 fluorescence level similar to the above described action of 2-APB. However, because there is no data investigating CAR effects on TRPM6 ion current it is unclear whether a concordance exists with electrophysiological data similar to that for 2-APB.

The present study shows that acidification of the cell incubation medium also caused changes in the immunodetected TRPM6 and TRPM7 channels. For TRPM7 the changes produced by extracellular acidification are also in the same direction as those observed for channel current. In cardiac myocytes extracellular acidification has been shown to increase the current attributed to TRPM7 in the presence of divalent cations^12,14^, but to decrease the current in the absence of extracellular divalent cations^11,12^. We are not aware of any electrophysiological study testing the effect of extracellular acidosis on TRPM6 currents in the absence of extracellular divalent cations.

Our study also shows a concordance between effects of chronic disease on channel expression and on channel currents. Chronic ischemic disease, which is associated with an increase of channel current density in electrophysiological studies focusing on atrial cells^12^, was also associated with an increase in immunodetected channel protein expression in the present study (see Fig. 5). The observed difference between non-IHD and IHD is unlikely to be explained by the small age difference between the two groups of patients, especially since no significant difference was obtained between age groups (patients aged 50–65 vs. those aged > 65) within the same clinical condition (either non-IHD or IHD) for the level of TRPM6 and TRPM7 measured by immunofluorescence and by ELISA (see Supplementary Table S8). The larger levels of TRPM6 and TRPM7 with chronic ischemic disease persisted for each age group. However, due to the modest sample size of each age group, additional studies may be needed to confirm this lack of an effect of age on the level of the proteins.

The other cardiac pathology known to affect TRPM7 channel expression is AF, but was not investigated in the present study. *Trpm7* mRNA and/or protein levels have been reported to be up-regulated in AF patients^14,41^ and in an animal model of HF^42^. Here too, there has been controversy concerning the detection and regulation in the specific situation of the LV, since up-regulation^43^, down-regulation^23^, and no detection^26^ of *Trpm7* mRNA expression levels were reported.

The role of cardiomyocyte TRPM7 and TRPM6 channels in cardiac pathology remains unclear. TRPM7 in cardiac fibroblasts has been implicated in cardiac inflammation and fibrosis as well as in atrial fibrillation, but the channel-enzyme has been considered either as a deleterious^41^ or as a protective factor^9^. Increased cardiac TRPM7 has also been associated with increased propensity to ventricular arrhythmia^43^. Based on findings in neurons, where TRPM7 mediates Ca^2+^ influx and cell death during ischemia^44^ it could be postulated that a similar mechanism operates in cardiomyocytes and contributes to cell death (infarction) during myocardial ischemia and/or reperfusion. The effect of acute ischemia on cardiomyocytes TRPM7 and TRPM6 remains unknown (but see^42^). For chronic ischemia, increased expression of the channel has been reported as mentioned above^12^. As far as reperfusion is concerned, instead of an activation of TRPM7 in cardiomyocytes by reactive oxygen species, these have been shown to inhibit TRPM7^45^. In contrast, others found an increased expression of TRPM7 under conditions of prolonged exposure to oxygen stress^46^.

The above-mentioned concordance between effects of various experimental and pathological factors on immunodetected channel protein on the one side and on channel current on the other side adds to the difficulty in interpreting the action of these factors in electrophysiology experiments. Many of the electrophysiological changes induced by these factors can be obtained within seconds, making it unlikely that they involve channel density changes. However, instead of consisting of purely biophysical mechanisms involving the channel pore, effects recorded over many minutes could also involve, based on the results of the present study, a change in channel protein density in the membrane. It is not known whether the various factors may influence channel turnover between intracellular and extracellular pools. For example, the effect of 2-APB might be accounted for if its binding were to promote channel internalization of one channel protein but to inhibit that of the other.

One could also envisage the possibility that changed immunodetected TRM6 and TRPM7 in cells incubated with 2-APB or CAR results from protein conformations induced by these drugs, with an effect of the new conformations on the binding of antibodies. However, this cannot be a satisfactory explanation for the changed expression in ischemic myocardium. For IHD, probably a real change in protein expression occurs. Extracellular acidification is noted to acutely increase current through TRPM6 or TRPM7 channels by decreasing the sensitivity of the channel to block by divalent cations and increasing the permeability of monovalent cations such as Na^+^ and probably H^+^ itself^47^. It is assumed that protons and Ca^2+^ simply compete for a common site from where blocking effect is exerted by Ca^2+^ but not H^+^. If our hypothesis to explain the changed expression is correct, then the effect of incubating with higher proton concentration is more complex, involving also a change in the channel protein conformation.

In summary, this study provides the first demonstration of co-expression of both TRPM6 and TRPM7 proteins and genes in the four chambers of the human heart. Our findings also suggest that a disease condition such as IHD could increase their expression, and this might indicate that TRPM7 as well as the TRPM6 are involved in the pathophysiology of cardiovascular diseases. TRP channel blockers such as 2-APB and CAR oppositely affected the levels of immunodetected proteins, i.e., both of them reduced immunofluorescence of TRPM7 but increased such of the TRPM6.

## Materials and methods

### Ethics

The study was carried out in accordance with the European Community guiding principles outlined in the Declaration of Helsinki, and was approved by the Ethics Committee of Biomedical Research of Kaunas Region, Lithuania (No.2R-1344 (2.6–1), 23 February 2018).

### Patients

We used cardiac tissue specimens resected from patients (n = 72) undergoing open-heart surgery or from hearts removed from patients (n = 9) receiving heart transplantation.

Right atrial (RA) tissues were obtained during heart surgery for coronary artery bypass graft (n = 32) or valve surgery (n = 25) or both (n = 15) at the Hospital of the Lithuanian University of Health Sciences (LUHS). The overall characteristics of the open heart surgery patients are summarized in Table 1, which groups them depending on whether they had ischemic heart disease (IHD) (n = 47; of both sexes, with a mean age of 67.4 ± 1.4 years) or not (n = 25; of both sexes, with a mean age of 60.7 ± 2.4 years; *P* < 0.05 when comparing the ages of both groups). Written informed consent before surgical procedures was obtained. The specimens were transported from the hospital to the laboratory in cold (8–10 °C) St. Thomas cardioplegic solution (composition in mmol: 110 NaCl, 16 KCl, 1.2 CaCl_2_, 16 MgCl_2_, 5 glucose, 10 HEPES, pH adjusted to 7.4, with NaOH). A part of each sample was frozen for biobank storage.

**Table 1:** Clinical characteristics of the patients.

Patient data	Non-IHD	IHD
Age range (years)	37–82	50–87
Mean age (years) ± SEM	60.7 ± 2.4	67.4 ± 1.4
Female, n (%)	10 (40.0)	17 (36.2)
Male, n (%)	15 (60.0)	30 (63.8)
Total, n (%)	25 (100)	47 (100)
**Surgical intervention**		
Valve surgery, n (%)	25 (100)	0 (0)
CABG surgery, n (%)	0 (0)	32 (68.1)
CABG and Valve surgery, n (%)	0 (0)	15 (31.9)

*CABG*, coronary artery bypass graft; *IHD*, Ischemic heart disease.

We also used tissues from whole human hearts, which were explanted from patients (9 males, aged 55.3 ± 3.0 years, age range: 38–65 years) undergoing cardiac transplantation at the same Hospital. Informed consent for the use of the explants for research purposes was obtained before cardiac surgery. In addition, five hearts were obtained from subjects after traumatic death following traffic accident and served as control (5 males, aged 48.6 ± 8.5 years, age range: 29–75 years) for immunohistochemical studies. Clinical characteristics of the patients and control subjects are presented in Supplementary Table S7 online. The explanted hearts were transported in cold St. Thomas cardioplegic solution. Muscle tissue samples were excised from all four heart chamber walls. Transmural left ventricular samples (taken up to 3 mm away from epi- and endocardial surfaces) were dissected from the mid portion of the heart. Part of dissected samples was snap-frozen at − 80 °C for biobank storage.

### Cardiomyocyte preparation

Cardiomyocytes were isolated as previously described^12,13^. In short, a small tissue specimen (from left atrium (LA), right atrium (RA), left ventricle (LV) and right ventricle (RV)) was fine-cut in oxygenated nominally Ca^2+^-free Tyrode solution (see composition below) supplemented with 3 mg/mL 2,3-butanedione monoxime, which was washed out 2–3 times before enzyme application. The tissue chunks were transferred to a beaker with nominally Ca^2+^-free Tyrode solution supplemented with 1 mg/mL bovine serum albumin (BSA), 1 mg/mL collagenase (215 U/mg, type 2; Worthington Biochemical Corporation, Lakewood, NJ, USA), and 0.5 mg/mL protease (7–14 U/mg, type XXIV; Sigma-Aldrich, St. Louis, MO, USA), and continuously bubbled with 100% O_2_. After 30 min of shaking in a water bath at 37 °C, the solution with both enzymes was replaced by fresh solution containing only collagenase (1 mg/mL), and shaken until cardiomyocytes appeared in aliquots obtained from the mixture. When the yield appeared to be optimal, the leftover of tissue chunks were resuspended in nominally Ca^2+^-free Tyrode and gently subjected to trituration by suction with a pipette. The cell suspension was filtered, centrifuged, and washed 2–3 times either with normal (divalent cation-containing) or with divalent cation-free Tyrode solution, and stored at room temperature. Dissociated cells were used for immunofluorescence studies.

### Immunofluorescence

The methods used for the immunofluorescence were similar to those described in detail previously^12^. Enzymatically dissociated cardiomyocytes were allowed to settle on the bottom of 8-chamber slides for 15–20 min. Afterwards, the cells were covered with hand-made perforated agarose coverslips, fixed in 4% paraformaldehyde for 15 min and then washed in physiologic buffer solution (PBS). Triton X-100 (0.1%) was used during 3 min for permeabilisation. Non-specific binding of antibody was prevented by using blocking buffer containing 10% bovine serum (Thermo Fisher Scientific, Waltham, MA, USA) in PBS for 1 h. Cells were incubated with primary rabbit polyclonal anti-TRPM6 antibody (ACC-046, Alomone labs, Jerusalem, Israel) or rabbit polyclonal anti-TRPM7 antibody (ACC-047, Alomone labs, Jerusalem, Israel) diluted (1:200) in PBS containing 3% BSA in blocking buffer overnight at 4 °C, and washed thereafter with PBS. For negative controls, incubation with primary antibody was omitted to check for non-specific binding of the secondary antibody. The cells were incubated for 1 h with fluorescence-labelled secondary antibody (either donkey anti-rabbit IgG; Alexa Fluor 488 conjugate; A21206, Invitrogen, Thermo Fisher Scientific, Rockford, IL, USA; dilution 1:200; or goat anti-rabbit IgG; Alexa Fluor 546 conjugate; A11035, Invitrogen, Thermo Fisher Scientific, Waltham, MA, USA; dilution 1:200), co-stained (for 20 min) with Phalloidin-Alexa Fluor 546 (A22283, Invitrogen, Thermo Fisher Scientific, Waltham, MA; dilution 1:100) or Phalloidin-CF 405 (00,034, Biotium, Fremont, CA, USA; dilution 1:100) and with Hoechst 33,342 (B2261, Sigma-Aldrich, St. Louis, MO, USA; 25 µg/mL; for 10 min) for labelling of the F-actin cytoskeleton and of the nucleus, respectively. We also used conjugated antibodies: mouse monoclonal anti-TRPM6 conjugate with AF546 (sc-365536, Santa Cruz Biotechnology, Inc., Dallas, TX, USA) and rabbit polyclonal anti-TRPM7 conjugate with AF488 (bs-9044R-A488, Bioss Antibodies, Woburn, MA, USA). Finally, the agarose covering and media chambers were removed and glass slides covered with ProLong Gold Anti-fade Reagent (P36934, Molecular Probes, Thermo Fisher Scientific, Waltham, MA, USA) and coverslip glass, and sealed with clear nail polish. Cardiomyocytes were visualized under confocal laser scanning microscope by utilising sixty-time magnifying (× 60) oil immersion objectives (Olympus BX61, Olympus Corporation, Tokyo, Japan) from which images were taken using the same scanning parameters (image acquisition control and spectral settings, etc.) for both TRPM6 and TRPM7 proteins in all cardiomyocyte preparations. Images are presented as stacks of 8–10 slices at fixed intensity. Cardiomyocyte area (pixels) and fluorescence intensity were measured in stacks using ImageJ 1.43 m (Wayne Rasband, National Institutes of Health, USA; http://rsb.info.nih.gov/ij) and Imaris 7.2.1 (Bitplane AG, Zurich, Switzerland; https://imaris.oxinst.com) softwares. Immunodetected TRPM6 and TRPM7 protein levels were calculated by the formula: fluorescence intensity*1000/cell area. In order to reduce any statistical confounder, immunofluorescence reading was blinded as the conditions used to keep cells were unknown to the person performing the reading.

### Preparation of homogenates from human heart tissues

We prepared tissue homogenates from all heart chamber walls. Small specimen were fine-cut in a dish on ice and placed in round-bottom centrifuge tubes. Ice-cold RIPA lysis buffer (150 mmol NaCl; 50 mmol Tris–HCl; 0.1% Triton X-100; 0.1% sodium dodecyl sulphate; 0.5% sodium deoxycholate) was added (~ 300 µL for ~ 5 mg of tissue) and the content was homogenized (LabGEN 125 Homogenizer, Cole-Parmer, Vernon Hills, IL, USA). Then 10 µL of a protease inhibitor cocktail were added and the homogenates was left on an orbital shaker for 2 h on ice. Afterwards the homogenate was centrifuged (Heraeus Biofuge Stratos Centrifuge, Thermo Fisher Scientific, Waltham, MA, USA) for 20 min at 10,000 rpm and 4 ºC, and the supernatant aspirated and placed in new tubes on ice. Pellets were discarded. The total protein concentration in the samples was determined using the Biuret Method. Homogenates were used for ELISA.

### Enzyme-linked immunosorbent assay (ELISA)

ELISA kits (MyBioSource, Inc., San Diego, CA, USA) were used to determine quantitative levels of TRPM6 and TRPM7 in human tissue homogenates. This method was performed based on the manufacturer’s protocol. We prepared samples and different concentrations of the standard. One well was blank. In total, 100 µL of each standard and samples were added to the appropriate microtiter plate wells with a biotin-conjugated antibody preparation specific for TRPM6 or TRPM7. The enzyme–substrate reaction was terminated by the addition of sulphuric acid. Optical signals were recorded with a spectrometer (Multiskan MF, Thermo Fisher Scientific, Vantaa, Finland) at a wavelength of 450 ± 10 nm. The levels of TRPM6 and TRPM7 were determined according to instructions provided by the ELISA kits: TRPM6 (MBS457214) and TRPM7 (MBS457216). After measuring the optical densities of the standard solutions, a standard curve was drawn using the Curve Expert 1.4 software (Daniel G. Hyams, Hyams Development, Chattanooga, TN, USA; https://curveexpert.software.informer.com/1.4). The concentrations of TRPM6 and TRPM7 in the tissue homogenates were then determined by comparing the optical density of the samples to the standard curve. The measurement of each single sample was read three times.

### Gene expression

RT-qPCR was used to detect the mRNA levels of *Trpm6* and *Trpm7* in heart muscles obtained from four chamber walls of three explanted hearts (twelve samples of total human RNA). The total RNA was isolated using the mirVana miRNA Isolation Kit (Thermo Fisher Scientific, Vilnius, Lithuania) and acid phenol:CHCl_3_ premix (Ambion, Austin, TX, USA) in accordance with the manufacturer ‘s instructions. An additional RNase Inhibitor (Applied Biosystems, Waltham, MA, USA) was added to the lysis buffer. RNA integrity was assessed using a 2100 Agilent Bioanalyzer System (Agilent Technologies Inc., Santa Clara, CA, USA). Prior to the start of cDNA synthesis, DNA contamination was removed from RNA samples using the TURBO DNA-free Kit (Thermo Fisher Scientific, Vilnius, Lithuania). Reverse transcription reactions were completed using a High Capacity RNA-to cDNA Kit (Thermo Fisher Scientific, Vilnius, Lithuania). cDNA was further diluted three times with nuclease-free water. After reverse transcription, the reaction mixture (cDNA) was used for qPCR. The qPCR reaction was done using TaqMan Assays (assays ID: Hs01019356_m1, Hs00559080_m1, Hs99999903_m1; Thermo Fisher Scientific, Waltham, MA, USA), TaqMan Universal MasterMix II, with UNG (Thermo Fisher Scietific, Waltham, MA, USA) and Nuclease-Free Water (Ambion, Austin, TX, USA). qPCR was performed by an ABI 7900HT Fast Real Time PCR System (Applied Biosystems, Waltham, MA, USA) based on the manufacturer’s procedure in 20-µL reaction mixture. *β-actin* (ACTB) was used as the endogenous control. The relative expression of target genes was calculated using the ΔCt method (2^-ΔΔCt^). ΔCt = Ct_target gene_ − Ct_β-actin_, where Ct refers to the number of amplification cycles when the real-time fluorescence intensity reached the set threshold whereby the amplification procedure was in the logarithmic growth phase. Each reaction was carried out in triplicate.

Gel electrophoresis of PCR products was used to analyse reaction quality and yield. A 1% agarose gel was prepared using Agarose (Cleaver Scientific, Rugby, Warwickshire, UK), 1 × TAE electrophoresis buffer (Thermo Fisher Scietific, Waltham, MA, USA), and 0.5 μg/mL ethidium bromide solution. PCR product samples of 10 μL were loaded onto a gel together with a 1μL of marker TriTrack Loading Dye (Thermo Fisher Scietific, Waltham, MA, USA). Electrophoresis was performed under the following conditions: 45 min, 100 V. The GeneRuler Ultra Low Range DNA Ladder marker (Thermo Fisher Scietific, Waltham, MA, USA) was used in the study. The gel was analyzed under UV light using the BDA Digital UV System (Biometer, Gottingen, Germany).

### Immunohistochemistry

For histological analysis, specimens of formalin-fixed and paraffin-embedded human heart muscle tissues were sliced in 3 μm and placed onto Super Frost Plus slides (Menzel, Braunschweig, Germany). Collected tissue sections were deparaffinized and stained with hematoxylin–eosin (H & E) and Picro Sirius Red.

For immunohistochemical analysis, deparaffinized sections were washed with distilled water, and then the epitope was released by incubating the preparations in TRIS/EDTA buffer (pH 9.0) at 110 °C for 8 min using microwave tissue processor RHS-1 (Milestone Medical, Bergamo, Italy). Immunohistochemical staining was performed using Shandon Coverplate plates (Thermo Fisher Scientific, Waltham, MA, USA). The sections were incubated with primary rabbit polyclonal TRPM7 antibody (1:250 dilution; NBP1-20224, Novus Biologicals, Centennial, CO, USA) or primary mouse monoclonal TRPM6 antibody (1:50 dilution; sc-365536, Santa Cruz Biotechnology, Inc., Dallas, TX, USA) for 1 h after blocking endogenous peroxidase. The preparations were then processed with the DAKO EnVision Flex and DAKO EnVision Flex + visualization system (Agilent Technologies Inc., Wood Dale, IL, USA) for TRPM7 and TRPM6, respectively. Probed sections were additionally stained with Mayer’s hematoxylin and coated with cover glasses using a polystyrene coating material. Colon tumor tissue sections were used for positive immunohistochemistry control. The IgG of the same isotype as the primary antibody dilution solution served as negative control: rabbit IgG isotype control (MA5-16384, Invitrogen, Thermo Fisher Scientific, Waltham, MA, USA) for TRPM7, and mouse IgG1 kappa monoclonal isotype control (ab91353, Abcam, Cambridge, UK) for TRPM6.

The histopathological evaluation was performed using Olympus BX61 (Olympus Corporation, Tokyo, Japan) motorized microscope and Evolution QEi camera.

### Chemicals

Carvacrol (CAR, ≥ 97% purity) was purchased from Carl Roth GmbH + Co (Karlsruhe, Germany), 2-APB from Abcam (ab120124, Cambridge, UK), and other chemicals were from Sigma-Aldrich (St. Louis, MO, USA). Water-insoluble compounds were initially dissolved in dimethyl sulfoxide (DMSO) or ethanol to make stock solutions (100 mmol), which were then diluted. The highest concentration of the solvent was < 0.1% and did not affect the measurements.

### Statistics

Average data are presented as mean ± standard error of the mean (SEM). Means were compared using the two-tailed *t*-test or ANOVA with LSD test (Least Significant Difference) for evaluating differences between groups. *P* < 0.05 was taken as threshold for statistical significance.

## Supplementary Information

Supplementary Information.

## Data Availability

All data are included in this manuscript and in its Supplementary materials.
