# Supplementary material for: Evidence for the expression of TRPM6 and TRPM7 in cardiomyocytes from all four chamber walls of the human heart
Source: Sci Rep. 2021 Jul 29;11:15445. doi: 10.1038/s41598-021-94856-4 (PMC8322396; doi:10.1038/s41598-021-94856-4)
Supplement: Supplementary file 1 — Supplementary Information. [file 41598_2021_94856_MOESM1_ESM.docx]

**Supplementary Material**

**Evidence for the expression of TRPM6 and TRPM7 in cardiomyocytes from all four chamber walls of the human heart**

**Inga Andriulė^1^, Dalia Pangonytė^1^, Mantė Almanaitytė^1^, Vaiva Patamsytė^1^, Milda Kuprytė^1^, Dainius Karčiauskas^2^, Kanigula Mubagwa^3,4^, Regina Mačianskienė^1,*^**

1. Institute of Cardiology, Lithuanian University of Health Sciences, Kaunas, Lithuania
2. Department of Cardiac, Thoracic and Vascular Surgery, Hospital of Lithuanian University of Health Sciences Kauno Klinikos, Lithuanian University of Health Sciences, Kaunas, Lithuania
3. Department of Cardiovascular Sciences, Faculty of Medicine, K U Leuven, Leuven, Belgium
4. Department of Basic Sciences, Faculty of Medicine, Université Catholique de Bukavu, Bukavu, DR Congo

*Corresponding author [regina.macianskiene@lsmuni.lt](mailto:regina.macianskiene@lsmuni.lt)

**Supplementary data**

Gel electrophoresis of PCR products was used to analyse reaction quality and yield. A 1% agarose gel was prepared using agarose (Cleaver Scientific, Rugby, Warwickshire, UK), 1x TAE electrophoresis buffer (Thermo Fisher Scietific, Waltham, MA, USA), and 0.5 μg/mL ethidium bromide solution. PCR product samples of 10 μL were loaded onto a gel together with a 1μL of marker TriTrack Loading dye (Thermo Fisher Scietific, Waltham, MA, USA). Electrophoresis was performed under the following conditions: 45 min, 100 V. The GeneRuler Ultra Low Range DNA Ladder marker (Thermo Fisher Scietific, Waltham, MA, USA) was used in the study. The gel was analyzed under UV light using the BDA Digital UV System (Biometer, Gottingen, Germany).

**
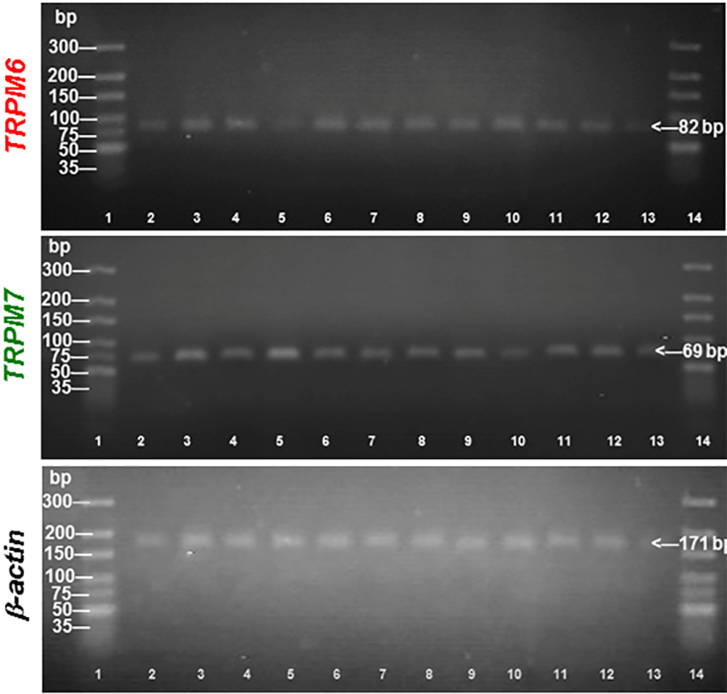
**

**Supplementary Figure S1.** TRPM7, TRPM6, and β-actin RT-qPCR images of human *Trpm6* and *Trpm7* genes detected in four cardiac chambers from three explanted human hearts: Lanes 1 and 14 from left to right contain molecular weight markers (GeneRuler-Ultra Low Range DNA Ladder, SM1213, Thermo Fisher Scientific, Waltham, MA, USA). Lanes 2-13 contain 82 and 69 base pairs of *Trpm6* and *Trpm7* PCR products*,* respectively (LA (lanes 2-4), RA (lanes 5-7), LV (lanes 8-10), and RV (lanes 11-13) for each explant).

**Supplementary Tables**

**Supplementary Table S1.** Immunofluorescence of TRPM7 and TRPM6 proteins in all cells used

| **TRPM7 DV** | **LA** | **RA** | **LV** | **RV** |
| --- | --- | --- | --- | --- |
| 2 h | 0.0900±0.0019  n =125 | 0.1046±0.0015#  n=120 | 0.0997±0.0023  n=58 | 0.1244±0.0027#  n=39 |
| 12 h | 0.1275±0.0020*  n=36 | 0.1365±0.0012*#  n=65 | 0.1481±0.0032*  n=24 | 0.1577±0.0058*  n=13 |
| **TRPM6 DV** |  |  |  |  |
| 2 h | 0.0541±0.0012  n=101 | 0.0590±0.0015#  n=124 | 0.0610±0.0019  n=77 | 0.0666±0.0029  n=55 |
| 12 h | 0.0771±0.0054*  n=26 | 0.0787±0.0018*  n=65 | 0.0859±0.0038*  n=35 | 0.0917±0.0063*  n=19 |
| **TRPM7 DVF** |  |  |  |  |
| 2 h | 0.0727±0.0013 n=68 | 0.0879±0.0011#  n=125 | 0.0772±0.0017  n=57 | 0.0975±0.0024#  n=25 |
| 12 h | 0.1170±0.0014*  n=60 | 0.1237±0.0023*#  n=36 | 0.1215±0.0019*  n=35 | 0.1402±0.0026*#  n=34 |
| **TRPM6 DVF** |  |  |  |  |
| 2 h | 0.0387±0.0024  n=37 | 0.0421±0.0014  n=119 | 0.0442±0.0019  n=76 | 0.0430±0.0033  n=24 |
| 12 h | 0.0454±0.0016†  n=69 | 0.0683±0.0033*#  n=70 | 0.0631±0.0021*  n=61 | 0.0749±0.0036*#  n=37 |

TRPM7 and TRPM6 – transient receptor potential melastatin type 7 and 6 channels; LA – left atrium, RA – right atrium, LV – left ventricle, and RV – right ventricle; DV – divalent cations; DVF – divalent cations-free, h – hour; n – number of cells; *P < 0.001 and † P < 0.05 for 2 h vs. 12 h; #P *<* 0.05 for right-sided vs. to left-sided.

**Supplementary Table S2.** Immunofluorescence results of conjugated vs. non-conjugated TRPM7 and TRPM6 antibodies

| **TRPM7**  DV, 2 h | **LA** | **RA** | **LV** | **RV** |
| --- | --- | --- | --- | --- |
| Conj. Ab | 0.0706±0.0004  n=7 | 0.0840±0.0004#  n=21 | 0.0866±0.0008  n=4 | 0.0972±0.0017#  n=7 |
| Non-conj. Ab | 0.0796±0.0005*  n=100 | 0.0978±0.0005*#  n=100 | 0.093±0.0005*  n=51 | 0.1137±0.0007*#  n=27 |
| **TRPM6**  DV, 2 h |  |  |  |  |
| Conj. Ab | 0.0371±0.0005  n=7 | 0.0424±0.0001#  n=21 | 0.0451±0.0004  n=4 | 0.0479±0.0003#  n=7 |
| Non-conj. Ab | 0.0504±0.0003*  n=92 | 0.0519±0.0003*#  n=105 | 0.0541±0.0006*  n=65 | 0.0553±0.0005*  n=43 |

Conj. Ab – primary antibodies conjugated with secondary antibodies; Non-conj. Ab – not conjugated antibodies; *P < 0.001 for conj. Ab vs. non-conj. Ab; #P < 0.001 for right-sided vs. to left-sided. Other notations are the same as in Supplementary Table S1.

**Supplementary Table S3.** Immunofluorescence results in cardiomyocytes from patients with a clinical history of ischemic heart disease (IHD)

| **TRPM7**  **DV** | **LA** | **RA** | **LV** | **RV** |
| --- | --- | --- | --- | --- |
| 2 h | 0.1320±0.0009  n=25 | 0.1385±0.0003#  n=20 | 0.1447±0.0003  n=7 | 0.1485±0.0004#  n=12 |
| 12 h | 0.1589±0.0002*  n=6 | 0.1710±0.0008*#  n=6 | 0.1822±0.0017*  n=6 | 0.1946±0.0011*#  n=5 |
| **TRPM6**  **DV** |  |  |  |  |
| 2 h | 0.0924±0.0006  n=9 | 0.0982±0.0008#  n=19 | 0.0984±0.0007  n=12 | 0.1070±0.0003#  n=12 |
| 12 h | 0.1355±0.0013*  n=6 | 0.1440±0.0012*#  n=5 | 0.1468±0.0009*  n=6 | 0.1539±0.0009*#  n=5 |
| **TRPM7 DVF** |  |  |  |  |
| 2 h | 0.0982±0.0009  n=9 | 0.1132±0.0004#  n=22 | 0.1094±0.0008  n=7 | 0.1203±0.0002 #  n=5 |
| 12 h | 0.1491±0.0004*  n=7 | 0.1640±0.0009*#  n=5 | 0.1626±0.0013*  n=6 | 0.1800±0.0005*#  n=7 |
| **TRPM6 DVF** |  |  |  |  |
| 2 h | 0.0680±0.0005  n=7 | 0.0755±0.0004#  n=18 | 0.0785±0.0005  n=14 | 0.0842± 0.0007#  n=6 |
| 12 h | 0.1066±0.0012*  n=6 | 0.1164±0.0008*#  n=17 | 0.1213±0.0016*  n=6 | 0.1297±0.0012*#  n=5 |

*P < 0.001 for 2 h *vs.* 12 h; #P <0.05 for right-sided vs. to left-sided. Other notations are the same as in Supplementary Table S1.

**Supplementary Table S4.** Immunofluorescence results in cardiomyocytes from patients without clinical history of ischemic heart disease (non-IHD)

| **TRPM7**  **DV** | **LA** | **RA** | **LV** | **RV** |
| --- | --- | --- | --- | --- |
| 2 h | 0.0796±0.0005  n=100 | 0.0978±0.0005#  n=100 | 0.0935±0.0005  n=7 | 0.1137±0.0007#  n=27 |
| 12 h | 0.1236±0.0006*  n=32 | 0.1342±0.0004*#  n=61 | 0.1413±0.0005*  n=20 | 0.1467±0.0005*#  n=10 |
| **TRPM6**  **DV** |  |  |  |  |
| 2 h | 0.0504±0.0003  n=92 | 0.0519±0.0003#  n=105 | 0.0541±0.0006  n=65 | 0.0553±0.0005#  n=43 |
| 12 h | 0.0717±0.0004*  n=20 | 0.0755±0.0004*#  n=62 | 0.0781±0.0002*  n=31 | 0.0801±0.0002*#  n=16 |
| **TRPM7 DVF** |  |  |  |  |
| 2 h | 0.0688±0.0009  n=59 | 0.0825±0.0005#  n=103 | 0.0727±0.0005  n=50 | 0.0918±0.0009 #  n=20 |
| 12 h | 0.1147±0.0009*  n=56 | 0.1200±0.0011*#  n=33 | 0.1190±0.0009*  n=33 | 0.1349±0.0005*#  n=30 |
| **TRPM6 DVF** |  |  |  |  |
| 2 h | 0.0319±0.0006  n=30 | 0.0361±0.0006#  n=101 | 0.0365±0.0006  n=62 | 0.0371± 0.0004#  n=21 |
| 12 h | 0.0427±0.0003*  n=66 | 0.0528±0.0005*#  n=53 | 0.0590±0.0008*  n=57 | 0.0663±0.0005*#  n=32 |

*P < 0.001 for 2 h vs. 12 h; #P < 0.001for right-sided vs. to left-sided. Other notations are the same as in Supplementary Table S1.

**Supplementary Table S5.** ELISA results in cardiomyocytes of IHD vs. non-IHD patients

| **TRPM7** | **LA** | **RA** | **LV** | **RV** |
| --- | --- | --- | --- | --- |
| Non-IHD | 618.76±19.73  n=3 | 980.99±55.65#  n=16 | 712.05±29.92  n=13 | 821.75±35.14  n=5 |
| IHD | 889.69±9.9*  n=3 | 1470.81±47.51*#  n=31 | 1125.87±22.8*  n=12 | 1318.35±52.23*#  n=5 |
| **TRPM6** |  |  |  |  |
| Non-IHD | 174.57±14.16  n=3 | 276.49±17.25#  n=16 | 248.66±8.07  n=13 | 281.73±8.31  n=5 |
| IHD | 228.47±11.26  n=3 | 337.45±12.91*#  n=31 | 302.88±9.33*  n=12 | 363.68±16.48*#  n=5 |

ELISA – the enzyme-linked immunosorbent assay; IHD – ischemic heart disease; *P < 0.05 for Non‑IHD vs. IHD; #P < 0.05 for right-sided vs. left-sided using ANOVA. Other notations are the same as in Supplementary Table S1.

**Supplementary Table S6.** *TRPM6-* and *TRPM7-*specific mRNA expression

| RT-qPCR | LA | RA | LV | RV |
| --- | --- | --- | --- | --- |
| *TRPM7* | -2,91±0.29 | -2,76±0.43 | -1.42±0.31 | -0.68±0.31# |
| *TRPM6* | -4,68±0.50* | -4.32±0.42* | -4.04±0.28* | -3.74±0.37* |

RT-qPCR – the Real-time quantitative polymerase chain reaction; *P < 0.001 for TRPM7 vs. TRPM6; #P < 0.05 for right-sided vs. left-sided (n = 3 for each). Other notations are the same as in Supplementary Table S1.

**Supplementary Table S7** Explanted and control human hearts data

| # | Gender (M/F) | Age (Years) | Diagnosis | EF (%) | Left ventricular assist device implantation before heart transplantation in months |
| --- | --- | --- | --- | --- | --- |
| 1 | M | 65 | IHD | 15 | Implanted for 3 months |
| 2 | M | 53 | IDCM | 22 | Implanted, but is unclear for how long |
| 3 | M | 48 | PPH | 55 | Not implanted (right ventricular overload) |
| 4 | M | 59 | IHD | 16 | Implanted for 13 months |
| 5 | M | 38 | IDCM  Heart transplantation 15 years ago.  Chronic heart rejection. | 25 | Implanted, but is unclear for how long |
| 6 | M | 56 | IDCM | 18 | Implanted for 15 months |
| 7 | M | 65 | IHD | 19 | Implanted for 3 months |
| 8 | M | 57 | IDCM | 24 | Implanted for 10 months |
| 9 | M | 57 | IHD | 19 | Implanted, but is unclear for how long |
| 10 | M | 29 | TA | - | - |
| 11 | M | 50 | TA | - | - |
| 12 | M | 41 | TA | - | - |
| 13 | M | 48 | TA | - | - |
| 14 | M | 75 | TA | - | - |

IHD – Ischemic heart disease; IDCM – Idiopathic dilated cardiomyopathy; PPH – Primary pulmonary hypertension; TA - Traffic (motor-vehicle) accident.

**Supplementary Table S8** TRPM6 and TRPM7 expression results in different age groups

|  | **Immunofluorescence (DV, 2h)** | | | **ELISA** | | |
| --- | --- | --- | --- | --- | --- | --- |
| **TRPM7** | **non-IHD** | | **IHD** | **non-IHD** | | **IHD** |
| < 50 years | 0.0799±0.0006  n_c/p_=32/1 | | - | 814.18±86.4  n_p_=1 | | - |
| 50-65 years | 0.0935±0.0008  n_c/p_=237/6 | | 0.1383±0.0011*  n_c/p_=62/5 | 871.11±80.21  n_p_=20 | | 1300.63±64.08*  n_p_=14 |
| > 65 years | 0.0939±0.0009  n_c/p_=15/4 | | 0.1391±0.0008*  n_c/p_=10/4 | 1041.80±143.58  n_p_=4 | | 1348.68±107.81  n_p_=30 |
| **TRPM6** |  |  |  |  |  |  |
| < 50 years | 0.0516±0.0006  n_c/p_=31/1 | | - | 191.26±16.58  n_p_=1 | | - |
| 50-65 years | 0.0524±0.0002  n_c/p_=260/6 | | 0.0971±0.0007*  n_c/p_=43/5 | 264.67±18.14  n_p_=20 | | 334.83±13.60*  n_p_=14 |
| > 65 years | 0.0548±0.0010  n_c/p_=21/4 | | 0.0958±0.0004*  n_c/p_=11/4 | 236.57±44.03  n_p_=4 | | 319.13±31.27  n_p_=30 |

Cardiac tissues and isolated cells harvested from the human heart were divided into two parts, in order to examine the levels of both TRPM6 and TRPM7. Mean levels of proteins detected by immunofluorescence (in a.u.) and by ELISA in samples grouped by age (<50, 50-65, >65 years) and also separated according to clinical diagnosis with and without IHD. n_c/p_ and n_p_ indicate the number of cells/patients and the number of patients, respectively. *P < 0.05 for non‑IHD vs. IHD using the two-tailed *t*-test.
